# Self-Assembled Decanethiolate Monolayers on Au(001): Expanding the Family of Known Phases

**DOI:** 10.1021/acs.langmuir.2c01356

**Published:** 2022-08-11

**Authors:** Martina Tsvetanova, Alexey G. Syromyatnikov, Thomas van der Meer, Arie van Houselt, Harold J. W. Zandvliet, Andrey L. Klavsyuk, Kai Sotthewes

**Affiliations:** † Physics of Interfaces and Nanomaterials, MESA+ Institute for Nanotechnology, University of Twente, P.O. Box 217, 7500AE Enschede, The Netherlands; ‡ Faculty of Physics, Lomonosov Moscow State University, Moscow 119991, Russian Federation

## Abstract

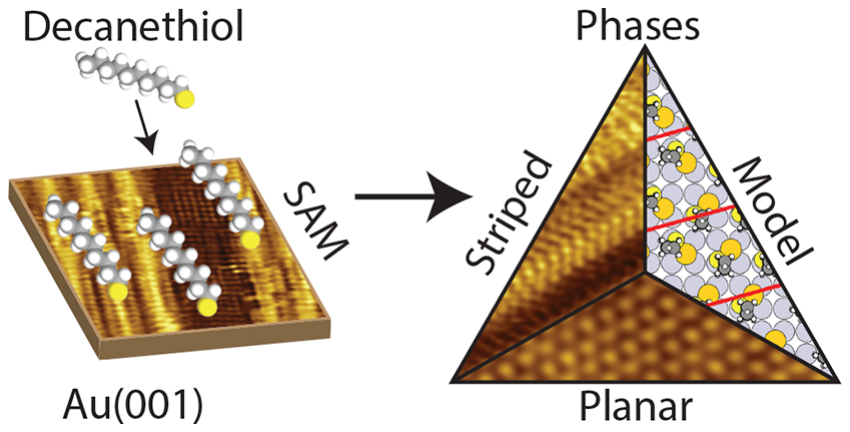

We have studied decanethiolate self-assembled monolayers on the Au(001) surface. Planar and striped phases, as well as disordered regions, have formed after exposing the Au surface to a decanethiol solution. The planar phases that we observe have a hexagonal symmetry and have not been previously reported for thiols on the Au(001) surface and have lower coverage compared to that of the other known thiol planar phases such as the square α phase. The striped phases that we observe are similar to the previously reported β phase but still feature unit cells that cannot be modeled as the archetype, and the coverage is also somewhat lower. The disordered decanethiolate regions are more dynamic compared to the ordered phases, confirmed with *I*(*t*) spectroscopy. This suggests that in these regions the coverage is too low in order to form ordered decanethiolate phases. Our findings contribute to the growing family of possible decanethiol formations on the Au(001) surface, for which still less is known compared to the extensive overview present for the Au(111) surface.

## Introduction

Self-assembled monolayers (SAMs) have already been proven useful in the field of nanotechnology and molecular electronics as good lubricants, corrosion inhibitors, and promising structural elements for various biomolecular devices.^1,2^ The formation and the properties of SAMs on various substrate terminations are, therefore, also interesting from a fundamental point of view. The most frequently used substrate termination in the case of Au is the three-fold symmetric Au(111) surface. At the same time, the other Au surfaces are less well investigated, although these interfaces with their different symmetry are in particular relevant for the Au-nanoparticle field, since nanoparticles have various terminations,^3−5^ including {111} and {001} facets.

When a thiol SAM forms on Au(111), the thiol sulfur head is deprotonated, we speak of a thiolate (RS) monolayer formation. A widely accepted picture is that the S-head forms a S–Au complex with an Au adatom, and the resulting staple motif (either of the form RS–Au–SR or RS–Au–SR–Au–SR) diffuses on the surface and eventually participates in the SAM formation.^3,6,7^ The Au adatoms are expelled from the surface due to the lifting of the herringbone reconstruction, additional Au atoms are also taken from the pre-existing step edges or from the terraces. That is why, the formation of vacancy islands on the terraces is a signature of thiol SAM formation for the thiol/Au(111) system.^7,8^ The self-assembly of molecular overlayers was widely researched in the case of Au(111) in terms of structure,^9−16^ dynamics and phase transitions,^17−23^ the ability of SAMs to tune the work function,^24−31^ and so forth. Numerous phases were reported for thiols on Au(111), the most well-known of which is the dense *c*(4 × 2) hexagonal phase,^9^ in which the molecular carbon backbones point upward at a small angle with respect to the surface normal. Striped phases of varying periodicity, generally coverage-dependent, were measured too, the striped nature of which arises due to the lying-down molecular tails.^10,13^

Bulk-truncated Au(001) crystals have square symmetry. However, in order to lower its energy, the Au(001) surface reconstructs into the so-called “hex” phase with a 25% higher atomic density compared to the unreconstructed surface.^32^ Throughout the past decades, this reconstruction has gathered a lot of attention in spectroscopic and diffraction studies,^32−37^ scanning tunneling miscroscopy (STM) and atomic force microscopy (AFM) studies,^37−42^ and in theoretical reports,^43−45^ to mention a few. Although there is a variety of reported unit cells, the accepted labeling formalism for the large hex unit cell is a (5 × *N*) structure, where *N* ∼ 20. The hex reconstruction resembles a striped phase made out of ribbons of width 5*a*, where *a* is the Au(001) lattice parameter of 0.288 nm. These ribbons feature a hexagonally distorted pattern (6 rows of this pattern fit on top of the 5 rows of the bulk-truncated substrate) and run along the  direction, or along the substrate steps. Small angle rotations up to 0.9° were reported for vicinal surfaces.^33,35−38^ The complexity of the Au(001) surface caused by the anisotropy of the hex overlayer makes Au(001) an interesting template to study the formation of self-assembled monolayers. In an electrochemical environment, the hex reconstruction is potential-dependent,^46^ which explains the interest in bare Au and SAM-covered electrodes in electrochemical cells,^46−53^ but apart from that, SAM formation on Au(001) was also studied in ultra high vacuum (UHV).^54−57^

Thiol SAM formation on Au(001) proceeds without the appearance of vacancy islands on the terraces. The hex reconstruction is, in general, lifted when a thiol SAM forms on Au(001). Considering that the hex overlayer is 25% denser than the bulk-truncated (001) surface, the formation of vacancy islands may not be necessary. It was shown by Grumelli et al. that an adatom bonding motif may still apply,^54^ considering the fact that nearby step edges appeared serrated. However, in a later work, the Au adatom motif was reported to be less likely.^55^ While for the Au(001) surface S can still deploy Au adatoms in sulfur–metal complexes, it seems that this is not always true for thiol molecules.^58,59^ In fact, it was shown that the bridging thiolate motif (−RS−) is more preferred than the staple motif for the Au(001) surface.^3^

When the hex reconstruction is lifted, generally monolayer-high Au islands form, covered by a continuous molecular phase. There are also striped regions, which mainly form at the locations where the hex reconstruction is not fully lifted. Taking into account the differences in the surface symmetry, the thiol molecular phases on Au(001) and on Au(111) are vastly different. Again, we can distinguish planar and striped phases on Au(001), but rarely phases in which the molecular carbon backbones are lying on the substrate. The striped nature of thiol phases on Au(001) is mainly a result of the arrangement of the Au adatoms underneath.

The first planar variant to mention is the nearly square α phase. Variations of this phase were measured in UHV in the case of hexanethiol,^54,55^ decanethiol,^56^ and in electrochemical environment for ethanethiol and propanethiol.^51,52^ The lattice parameter of the unit cell ranges between 0.4 and 0.5 nm, with the angle between the lattice vectors very close to 90°. The α phase grows on the regions of Au(001), where the reconstruction is lifted, at 45° with respect to the ⟨011⟩ direction, or the step edges. Although planar in nature, meaning that no Au adatoms are considered to be involved in the structure, the α phase shows striped variants, both in UHV and in an electrochemical environment. Grumelli et al.^55^ reported a ribbon-like  phase and a striped-like (2 × 7) pattern for hexanethiol on Au(001)-(1 × 1). The striped appearance was explained by the different relaxation of the surface Au atoms, bonded and not bonded to thiol molecules. Yamada and Uosaki^56^ reported a similar ribbon-like structure, incommensurate with the substrate, for decanethiol on Au(001)-hex. In an electrochemical environment, the α phase striped variant is an incommensurate (1.2*p*) structure, with *p* being either 7 or 8.2, measured for ethanethiol^51^ and confirmed to be very similar to the results for propanethiol.^52^ The reported α phase density in these studies^51,52,54−56^ varies between θ ∼ 0.36 and θ ∼ 0.44 ML, with respect to the bulk-truncated Au(001) surface.

Additional nonstriped molecular phases were reported for adlayers on Au(001), based on diffraction experiments. For instance, Dubois et al. suggested a *c*(2 × 2) structure for methylthiolate studied by low energy electron diffraction,^60^ also observed in the growth of sulfur adlayers on Au(001).^61^ The lattice parameter of this phase (4.07 Å), however, is smaller than the one for bulk alkanethiols. Therefore, the *c*(2 × 2) phase was considered unlikely to accommodate the molecular tails, although the authors suggested that the sulfur-substrate interactions may be important in bringing the molecular tails closer together compared to bulk alkanethiols. For docosyl mercaptan on Au(001), another phase of square symmetry was suggested based on electron diffraction experiments: an incommensurate *c*(10 × 10) structure with lattice parameter of 6.42 Å, oriented again at 45° with respect to the main crystallographic ⟨011⟩ direction.^62^ A later He diffraction study,^63^ however, has shown that both the *c*(10 × 10) and the *c*(2 × 2) structures are unlikely for docosyl mercaptan, which instead shows an incommensurate oblique structure with lattice parameters about 6 Å, and an angle about 95°.

Next, we look at a phase which is exclusively striped in nature, the so-labeled β phase, observed both in electrochemical environment and under UHV conditions. This is a hexagonally distorted molecular pattern with nonequal lattice parameters of the hexagonal unit cell between 0.4 and 0.58 nm and density θ ∼ 0.33.^52,54,56,57^ The β phase was explained as a molecular phase growing on 1 in 4 missing Au adatom row arrangement, or a *c*(2 × 8) structure, measured with STM for butanethiol and decanethiol in UHV conditions by Poirier.^57^ This explanation is widely accepted and the following reports adapted a similar explanation, or the deviations from this model were not discussed in detail.^52,54,56^ Loglio et al. reported an incommensurable structure instead.^53^ Grumelli et al.^54^ demonstrated the possibility that thiols can arrange on a still hexagonally ordered elongated Au adatom islands. Therefore, the β phase is not strictly defined, apart from the fact that the width of the β stripes closely resembles the width of the hex reconstruction ribbons. Thus, the β phase cannot be formed on the Au(001)-(1 × 1) surface. The surface to start with must be Au(001)-hex: the remainders of the hex reconstruction after the SAM is formed participate in the corrugation of the β phase, at least in the form of expelled Au adatom rows. There is no full consensus regarding the arrangement of these adatom rows. A *c*(2 × 8) structure was also observed with He diffraction for annealed n-octadecane SAMs on Au(001),^64^ but differently from the results of Poirier,^57^ a 1 × 4 added (instead of missing) Au adatom row was proposed as explanation for the corrugation, deduced by the observed periodicity.

Apart from the structure of SAMs on Au(001), their charge transfer properties^65^ and stability^66,67^ are gaining more attention. The electrochemical stability of thiols on Au(001) was shown to be higher as compared to thiols on Au(111): an effect shown to be larger for long-chain aliphatic thiols, and chain-length-independent for aromatic thiols, whereas the electrochemical stabilization for Se-based SAMs was even larger as compared to S-based SAMs.^66^ Thermal desorption experiments combined with X-ray photoemission spectroscopy^67^ confirm the view that SAMs of thiolates on Au(001) are more stable: for all experiments until *T* = 380 *K*, at a given moment, the total S-yield is higher for Au(001) compared to its Au(111) counterpart. Desorption already at room temperature in UHV is expected to transform the SAM landscape, as the coverage decreases. The desorption experiments presented in this paper also suggest the presence of lying-down phases on Au(001), which have not been observed elsewhere.

The overview presented here shows a rich variation in the molecular phases on Au(001). In this paper, we detail the structure of planar and striped decanethiolate arrangements which further expand the family of known phases. We keep the distinction between planar and striped phases as proposed in this overview. Furthermore, we also look at a disordered decanethiolate phase. Further studies are needed before a detailed classification of the phases can be made as for SAMs on Au(111).

## Methods

An Au(001) single crystal was used as a substrate (SPL, Zaandam, The Netherlands). The surface was cleaned by Ar^+^ sputtering cycles (ion energy of 1 keV and a time period per cycle between 20 and 45 min). The base pressure in the UHV system was <1 × 10^–9^ mbar, the Ar pressure was ∼3 × 10^–6^ mbar. After each sputtering cycle, the sample was annealed ex-situ in a quartz tube for 2 h at a temperature of 600 °C, under a constant N_2_ flow. After about a dozen cycles were performed, the Au(001) surface featured the expected hex reconstruction, measured with an RHK Technology STM at room temperature. The Pt–Ir STM tips were electrochemically etched from a wire. The SAMs were prepared in a solution. The Au(001) single crystal was submerged in a 1 mM 1-decanethiol (99%, Sigma-Aldrich) ethanolic solution for 1 h, just after the last annealing cycle. Afterward, the sample was rinsed with copious amount of ethanol and dried with N_2_. The freshly prepared sample was loaded in UHV conditions as soon as possible to avoid potential contamination. In all measurements, the STM tip is biased, while the sample is grounded.

Ab initio density functional theory (DFT) calculations of activation barriers for diffusion and interaction energy between molecules were performed by using the Vienna ab initio simulation package (VASP).^68,69^ The ion-electron interaction was described using the projector augmented-wave (PAW) technique.^70,71^ The GGA-PBE density functional^72^ was deployed. The PBE-GGA functional has been demonstrated to achieve a good balance between accuracy and computational effort for the decanethiol molecule on gold.^23^ We have used a plane wave basis set with a 400 eV kinetic energy cutoff. The substrate was modeled as periodically repeated slabs consisting of up to five atomic layers, separated by a sufficiently thick vacuum space. The bottom two layers of the slab are fixed at their bulk positions. The top three layers are allowed to relax upon optimization. The atomic positions were relaxed until the force on the unconstrained atoms was less than 0.03 eV Å ^–1^. We used a (4 × 8 × 1) Monkhorst–Pack k-point grid^73^ to sample the Brillouin zone of the surface unit cell. The contributions of van der Waals forces were estimated using dDsC dispersion corrections.^74^ To study the diffusion of a molecule on a gold surface, we employed the nudged elastic band method^75,76^ implemented in VASP to locate the saddle points of the potential energy surface and to search for the minimum energy pathway of diffusion. The minimum energy path was discretized by nine intermediate states between two states. The atomic models are drawn using ASE.^77^

To simulate the evolution of the system addressed in the computational results section and in the Supporting Information of this paper, we used the two-dimensional kinetic Monte Carlo (kMC) model similar to that described previously.^3,78−80^ The hop rate of Au–SR complex from site *i* to site *j* on Au(001) was calculated using the Arrhenius equation:
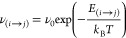
where *T* is the temperature of the substrate, ν_0_ is the attempt frequency, and *k*_B_ is the Boltzmann constant. We set ν_0_ to 10^12^ Hz. The influence of the interaction between Au–SR complexes on their diffusion was included in the activation barrier which takes the form^79^
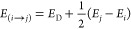
Here, *E*_*D*_ is the activation barrier for an isolated Au–SR complex on the clean surface. *E*_(*i*(*j*))_ is the sum of pair potentials due to the interaction between Au–SR complexes: the considered Au–SR complex with all the other complexes, if the Au–SR complex is at site *i*(*j*).

## Decanethiolate SAM on Au(001)

**Figure 1:**
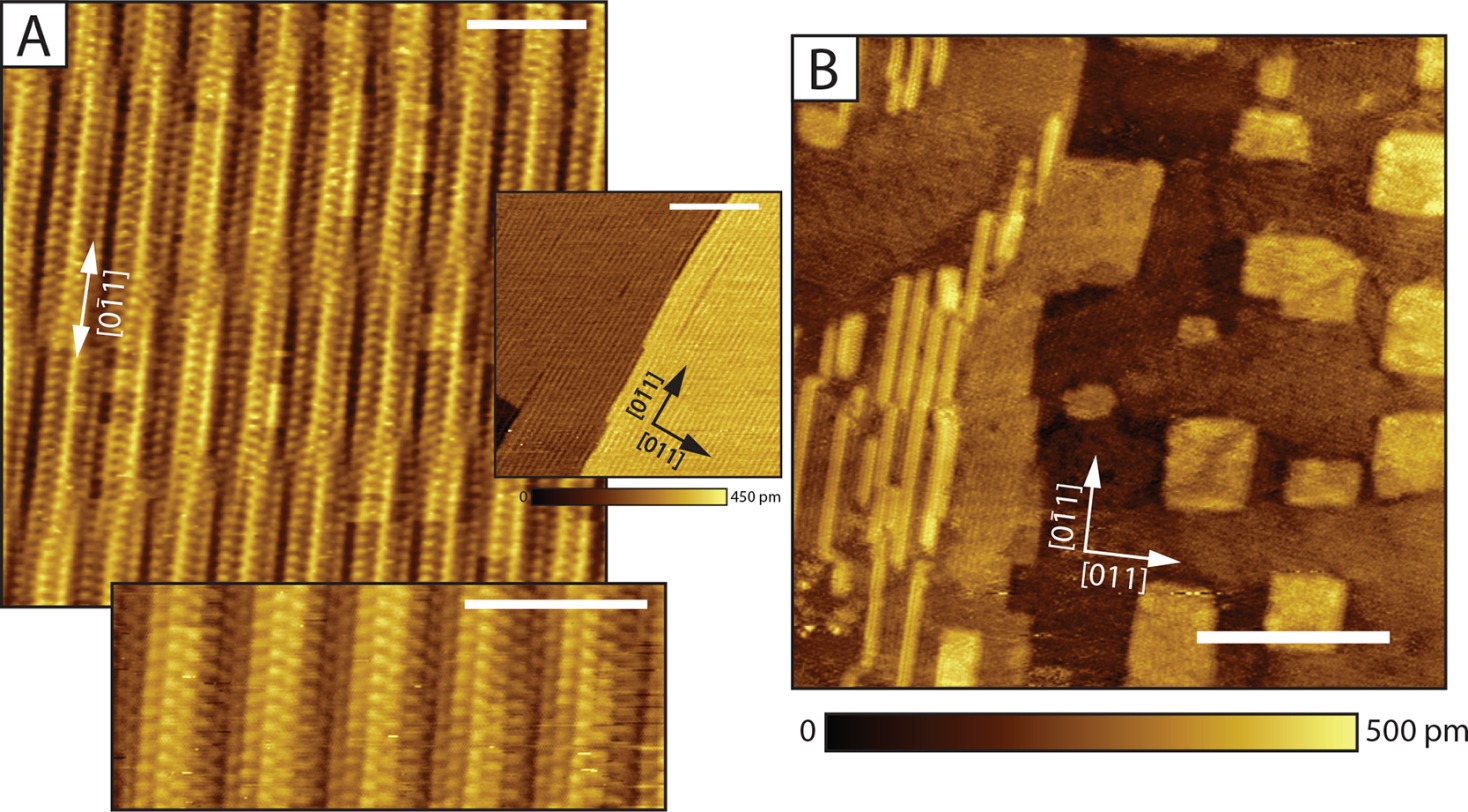
(A) STM data for the clean Au(001) surface. The main image is a 15 × 15 nm^2^ image of the hex reconstruction (set-points: −100 mV, −500 pA, scale bar: 3 nm). The small inset features a larger scale STM image, showing that the hex stripes are aligned with the ⟨011⟩ direction (set-points: −200 mV, −500 pA, scale bar: 20 nm). The lower inset shows an even more zoomed-in image of the hex phase (set-points: −90.0 mV, −500 pA, scale bar: 3 nm). Perpendicular to the hex stripe the width contains 6 Au atoms. (B) A 100 × 100 nm^2^ STM image of the SAM-covered surface (set-points: 0.5 V, 80 pA, scale bar: 30 nm). The hex reconstruction is lifted in most of the area. The surface features planar decanethiolate phases, monolayer-high Au islands, striped molecule phases, and disordered regions.

The clean Au(001) surface shows the expected hex reconstruction (Figure 1A). Bulk-truncated Au(001) crystals feature square symmetry. When the decanethiol SAM is formed, we can observe this symmetry as the reconstruction is partially lifted (Figure 1B). Because the hex-reconstructed surface has about 25% higher density compared to the bulk-truncated surface, square Au adatom islands form. Apart from Au adatom islands and planar regions, striped regions are observed, which preserve the former orientation of the hex reconstruction. At these striped phases, the hex reconstruction is most likely not fully lifted. The molecular SAM covers the surface, both at the planar and at the striped regions. Finally, we also observe disordered regions, in which no particular phase seems to form, nor the clean Au(001)-(1 × 1) surface is exposed.

## Phase Models

We present approximate models of the observed phases based on the bare thiol molecule. We have also attempted to model one of the observed phases with the Au–SR complex. To create the bare molecule models, we mostly assumed bridge and hollow adsorption sites. We avoided the unfavorable on Au(001) on-top position^3,55^ and assumed when possible the molecules to be in the preferred bridging thiolate motif (−RS−). The four-fold hollow sites were chosen as an alternative site as they are still more favorable than the on-top position: Hu et al.^3^ have also shown that when methylthiolate is placed on an atop position, a relaxation to a bridge site takes place, while for the square four-fold hollow sites, the magnitude of the binding energy is smaller, but comparable to the bridge site.

In our previous study,^81^ it was shown that the most likely diffusing moieties on Au(001) are still Au–SR complexes, in which a single decanethiol molecule binds to a Au adatom. Therefore, except simplified models inspired by the bridging motif, we performed a computational study as well, attempting to model one of the phases observed with Au–SR complexes instead of the bare molecule. The calculated interaction energies for two Au–SR complexes at different distances on top of the unreconstructed surface are given in Table 1. The calculation was performed as described in the methods. The model of the phase will be presented in the Computational Results section.

**Table 1:** Interaction Energies for Two Au–SR Complexes on the Surface As a Function of Relative Distance *r*/*r*_0_^a^

neighbor order	distance (*r*/*r*_0_)	*U*(*r*) (meV)
first	1	+297
second	2	+5
third	2	–114
fourth	5	–61
fifth	8	0

a *r*_0_ is the distance between the nearest gold atoms. The neighbor order is used to signify the growing distance.

For the striped phases, as explained later, added Au adatom rows are assumed, as well. The tolerances in the size of the Au(001) lattice parameters are discussed in the Supporting Information (SI) (see Table 1 in the SI). Stretching or contracting of the lattice parameters is assumed only within 10% of the expected Au(001)-(1 × 1) lattice spacing of 0.288 nm.

## Planar Decanethiolate Phases

A few planar decanethiol phases were observed. The structural models developed feature similarities and varying molecular coverage. All of these phases have lower coverage than the square α phase. We, therefore, suggest that these phases would form at the low coverage regime and if more decanethiolate molecules are available, they may transform into the denser square α phase.

### The φ Phase

**Figure 2:**
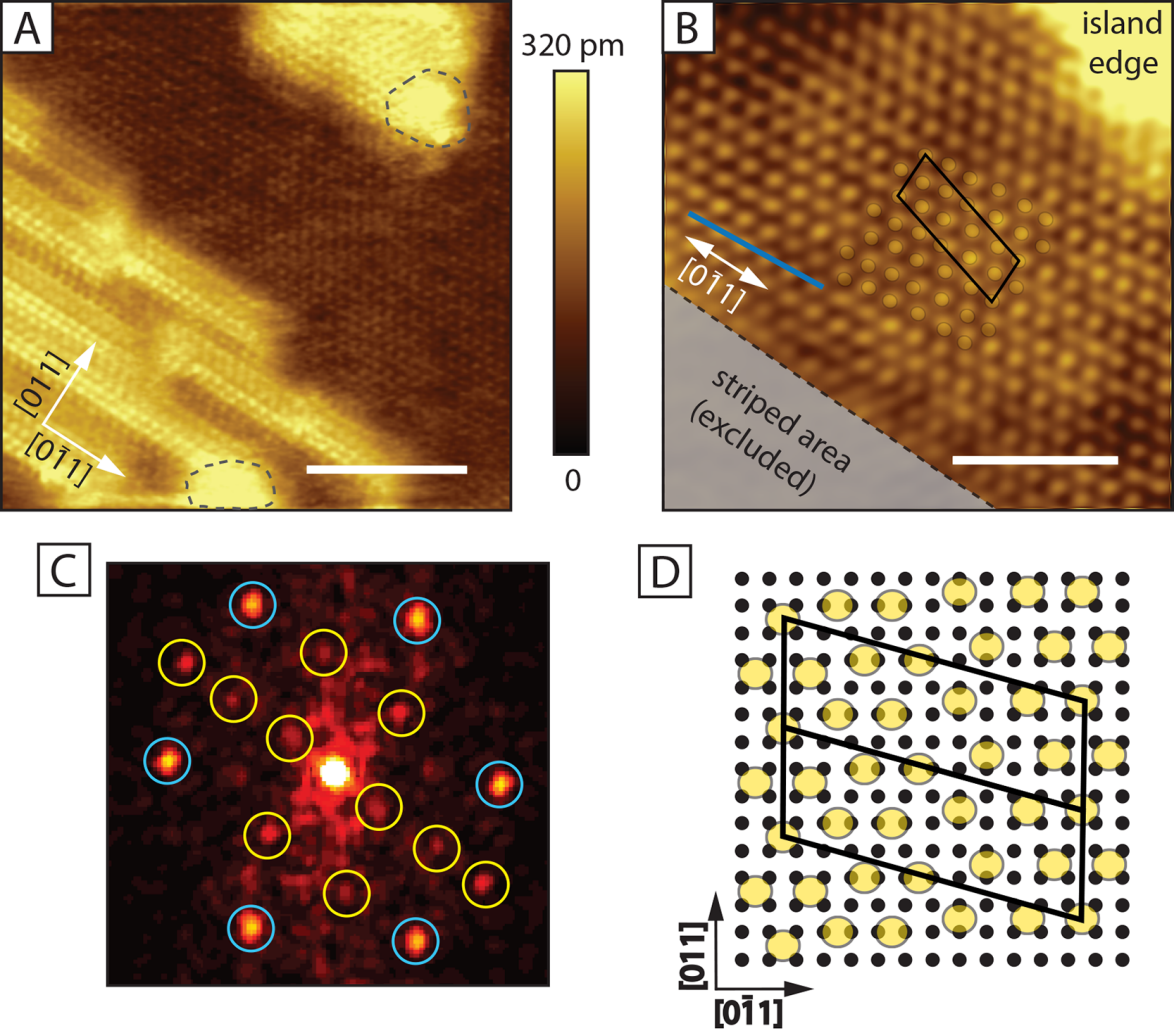
φ Phase. (A) 25 × 25 nm^2^ STM image of the φ phase (set-points: 0.5 V, 80 pA, scale bar: 8 nm), located between striped area and an island (also on top of the island). The dashed line encircles the dynamic features. (B) Zoomed-in 12 × 12 nm^2^ STM image (set-points: 0.5 V, 80 pA, scale bar: 4 nm). The unit cell is given in black, the model given in (D) is also overlaid on part of the data. A blue line indicates the orientation of the phase with respect to the main crystallographic direction, rotated at a small angle of 4–5°. (C) FFT pattern from the measurement in (B). The blue circles indicate the hexagonal structure, the yellow circles indicate the spots, part of the  overlay. (D) Proposed model for the φ phase. Two consecutive unit cells are marked in black. The yellow circles indicate the location of the S-head of the molecule. The small black circles correspond to the Au atoms in the topmost unreconstructed layer.

The first phase we address is a hexagonal phase, measured in a region between a stripe and an Au adatom island (see Figure 2). The islands are also covered with SAM phases, but the resolution on top is worse. Note the “fuzzy” appearance of the edges of the island and the stripes in Figure 2(A), as well as the bright marked features. This fuzzy appearance is due to real time changes of the structure under the STM tip. The structures are thus dynamic under the present circumstances.

In Figure 2B, we observe some distortions and large-scale height variations, this may imply that the phase features slight incommensurability with the Au substrate. However, some features are repeating and that is why we proceeded with the analysis. The unit cell we suggest in (D) is inspired by the Fourier transform (FFT) pattern in (C), corresponding to a  overlay. Not all of the spots are present in the FFT pattern, but their locations correspond to the expected for this overlay positions. We will refer to this phase as the φ phase, due to its hexagonal arrangement, in analogy to the hexagonal phase of thiols forming on Au(111).

Modeling the φ phase in real space with its  structure without observing exposed Au regions is a challenge. Nevertheless, overlying the expected Au(001)-(1 × 1) grid suggested that the observed phase features striking similarity to a *c*(2 × 4) structure with respect to the unreconstructed substrate. This structure was formerly observed for S adlayers on Au(001).^82^ Therefore, considering that the decanethiol molecules have S-heads, it is reasonable to assume that the SAM may feature similar regions. Next, due to the fuzzy appearance of the island and the stripe edges and the slight piezo drift, it may be hard to notice in the upper part of Figure 2A, but at the lower part of this image, as well as in Figure 2B, it becomes clear that the hexagonal phase is slightly rotated (4–5°) with respect to the main ⟨011⟩ crystallographic direction. Based on these: the similarity to the *c*(2 × 4) S-adlayer phase and the rotation, we propose the model in Figure 2D. Note that for the model there are 4 molecules in a hollow site and 6 molecules in a bridge position in each unit cell, thus still more molecules with a preferred bridging motifs.

The density of the φ phase according to the model is θ ∼ 0.23, slightly lower than the density of the *c*(2 × 4) phase (θ = 0.25) observed for S-adlayers.^82^ The reduced density can be easily explained by the fact that now the phase needs to accommodate the molecular tails too, a possible reason also for the slight rotation of this hexagonal phase. Note that we observed rotation with both positive and negative angle of a few degrees with respect to the ⟨011⟩ direction (see Figure S1 in the SI). The orientation of the molecular tails and their interactions with the STM tip may be responsible for deviations of the model from the true molecular arrangement. The use of low voltage bias in our experiments, however, helps in keeping the effect of the tails to a minimum. Due to the large band gap of thiols (a few eV), tunneling to or from HOMO/LUMO states in our configuration is not likely. Electrons must be tunneling through the tails to the adsorbed S atoms.

### The φ′ Phase

**Figure 3:**
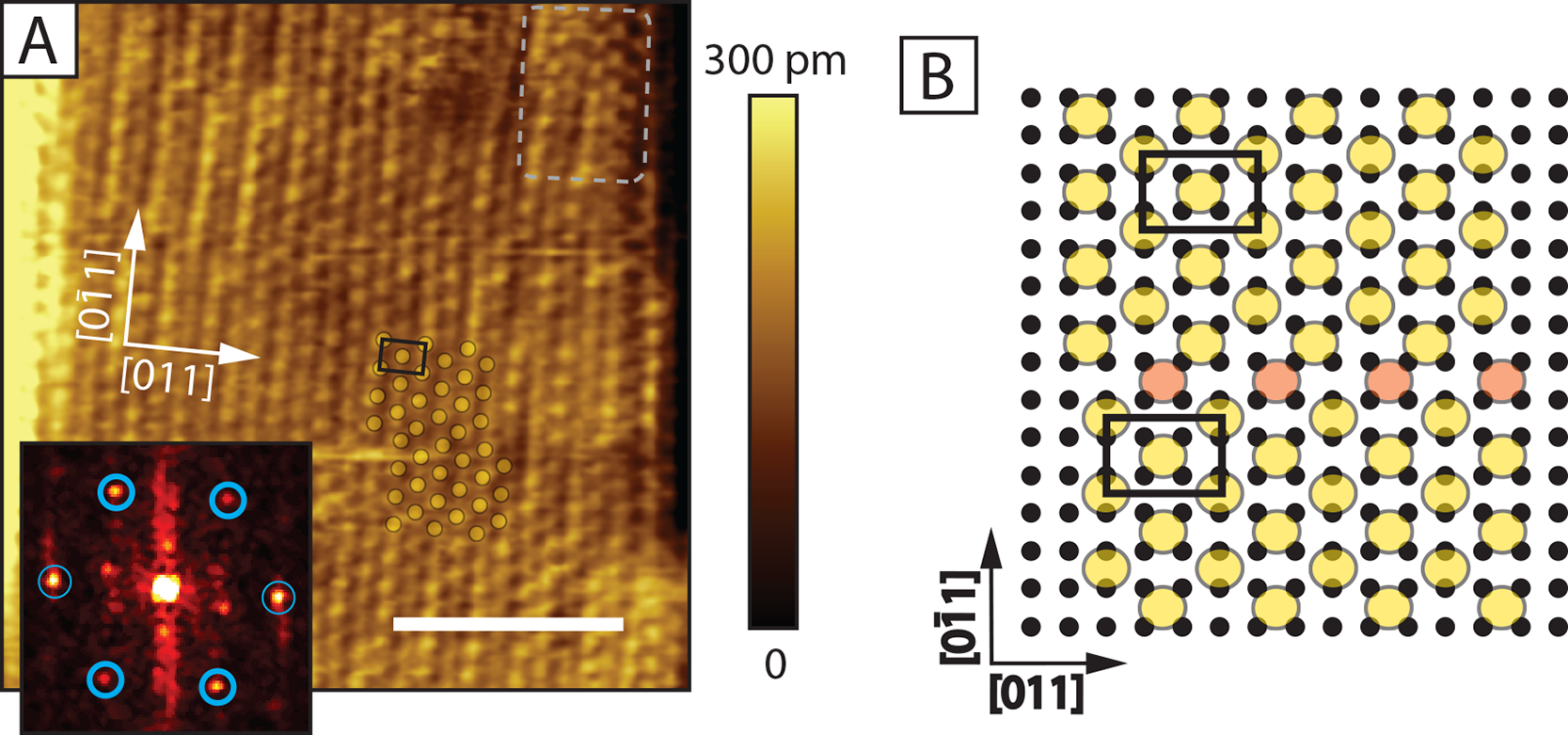
φ′ Phase. (A) 15 × 15 nm^2^ STM image of the φ′ phase (set-points: 0.5 V, 80 pA, scale bar: 5 nm). An FFT is shown as an inset. The circles indicate the hexagonal pattern, whereas the circles with thicker lines indicate the centered cell selected. The unit cell is marked also on the STM image in black. Part of the model shown in (B) is overlaid on part of the data. A dashed overlay indicates a striped region which has slightly different ordering in the top right corner. (B) Suggested model for the φ′ phase. Large circles indicate the location of the molecular S-heads, and small black circles indicate the Au(001)-(1 × 1) topmost layer. The *c*(2 × 3) unit cell is marked with a black rectangle, analogous to the unit cell in (A). Orange-colored circles indicate the location of an antiphase boundary. A shift in the opposite direction must be possible, too.

We continue with another hexagonal phase, that we will refer to as φ′. This phase features some small height variations, thus we treat this phase as a planar phase, in the sense that no Au adatom rows are involved in the structure. A STM image of the φ′ phase is shown in Figure 3A. The phase is positioned between a striped feature (left) and a step edge (right). From these we deduced the ⟨011⟩ direction and aligned the Au(001)-(1 × 1) grid. It is also visible from the image in Figure 3A that the φ′ phase consists of alternating brighter and darker rows and these rows are generally aligned with the step edges.

The FFT pattern of a defect-free part from the φ′ phase is shown in the inset of Figure 3A. We observe a hexagonal pattern, a centered rectangular cell can be selected. The corresponding centered cell is marked also in real space. Based on this unit cell, we propose a simplified model of the φ′ phase in Figure 3B. Rows of bridge positions and four-fold hollow sites alternate. The rectangular unit cell has a *c*(2 × 3) form.

Assuming that the molecules in bridge positions are slightly higher compared to the molecules in the hollow sites explains the alternating bright and dark rows in the STM image in Figure 3A. The darker colored circles in the model indicate an antiphase boundary: the φ′ phase shifts, resulting in transition of a bright row to a dark row and vice versa, as sometimes observed in the STM image. Another reason for possible molecular shifts and departure from this simplified view is the tendency of the molecules to relax to bridge positions. Moreover, once again we stress upon the fact that we cannot model the exact effect of the molecular tails orientation to the observed topography, nor the effects of potential slight incommensurability. Nevertheless, the latter can easily agree with the need of antiphase boundaries. Note that the antiphase boundary may appear at a bridge-site row and in opposite direction too, not in particular as shown in Figure 3B for a hollow-site row and shift to the left. In fact, when overlaying the model onto the data in (A), we assumed the shift appears at a bridge site row, followed by another bridge site row.

The coverage of this phase as deduced from the *c*(2 × 3) unit cell is θ = 0.33. Compared to the φ phase, this is a slightly higher coverage. Also now, the phase is not rotated with respect to the main crystallographic direction. Apart from that, the models suggested so far in Figure 2D and Figure 3B are very similar: the one in 2(D) was deduced by starting from a *c*(2 × 4) structure, while the one in 3(B) has a *c*(2 × 3) unit cell. It is possible that the slightly higher local SAM coverage leads to the formation of the φ′ phase instead of the φ phase, which is the abundant planar phase at the location in which it was measured.

The coverage of 0.3 < θ < 0.4 is frequently reported^55,57,64^ and is typical for the striped β phase variants as can be deduced from the suggested unit cells. This is also consistent with the nearly striped appearance of the φ′ phase. Note also the region enclosed with a dashed line in Figure 3A, where there is a clear departure from the structure of the surrounding phase. Although different, the region still resembles a striped phase. We chose to treat the φ′ phase as a planar phase because the height variations are not large enough to assume the presence of Au adatom rows in the overall structure, merely a question of classification. Nonetheless, striped variants even of the planar phases were reported earlier,^51,55^ regardless of the participation of Au adatoms: the different inward/outward relaxation of the Au atoms in the same monolayer can also lead to a striped appearance. Thus, it is not strange that the φ′ phase features striped appearances too. The lack of clear boundary between such domains of the φ′ phase suggests that there are more decanethiolate phases to be yet found, possibly very close structurally and energetically to the phases that we measured.

In relation to the current analysis, it is important to note that an additional variation of the φ′ phase was also measured. We labeled this phase as the φ^″^ phase. Due to the similarities between these phases, we assumed that they may just be rotational domains of the same structure. The φ^″^ phase is discussed in the Supporting Information, where a possible model is provided too (see Figure S2 of the SI).

In summary, we presented two hexagonal phases. The φ phase has a coverage of 0.23 ML, it is structurally similar to a *c*(2 × 4) phase, has a  overlay structure, and is slightly rotated with respect to the ⟨011⟩ direction (4–5°). The φ′ phase has a *c*(2 × 3) unit cell, a coverage of 0.33 ML, and features alternating higher and lower molecular rows. These phases are very similar to each other and are possibly energetically close too. Most likely the slightly higher local SAM coverage in the region of the φ′ phase leads to its formation instead the formation of the φ phase. Variations of the φ′ phase were measured as well, suggesting that there are yet more decanethiolate structures to be found in the low coverage regime, before the square α phase forms at 0.44 ML coverage.

It is also important to acknowledge the possibility that the SAM may rearrange in time, leading to the formation of the φ′ phase at the expense of the φ phase, as the latter was not spotted close to the former at all. That is an alternative view to the local coverage view, as it is virtually impossible to evaluate the local SAM coverage with great precision, especially due to the presence of disordered SAM regions. Although the time evolution of SAMs was mainly researched in electrochemical environment where the potential applied to the electrodes can be altered rather fast,^51^ Au rearrangement and coalescence of Au structures was reported also for systems in UHV, for which the experimental parameters are not altered or cycled.^57^ This, combined with the fact that thiols readily desorb in vacuum^67^ makes it hard to exclude this alternative scenario. We note that the generally lower SAM coverage in our experiments may further assist this process. In a denser SAM, the van der Waals tails interactions may also help in stabilizing better the phases and may reduce the dynamic effects.^66^ Therefore, more experiments with prolonged time monitoring will be needed in order to investigate further the relationship between the phases that we presented.

## Striped Decanethiolate Phases

In this section we will discuss phases which are exclusively striped in nature, meaning that Au adatoms are most likely also part of the overall structure. These phases resemble the previously reported β phase, that is why we have labeled them as the β′ phase and the β^″^ phase. None of these, however, can be modeled as the original archetype.

### The β′ Phase

**Figure 4:**
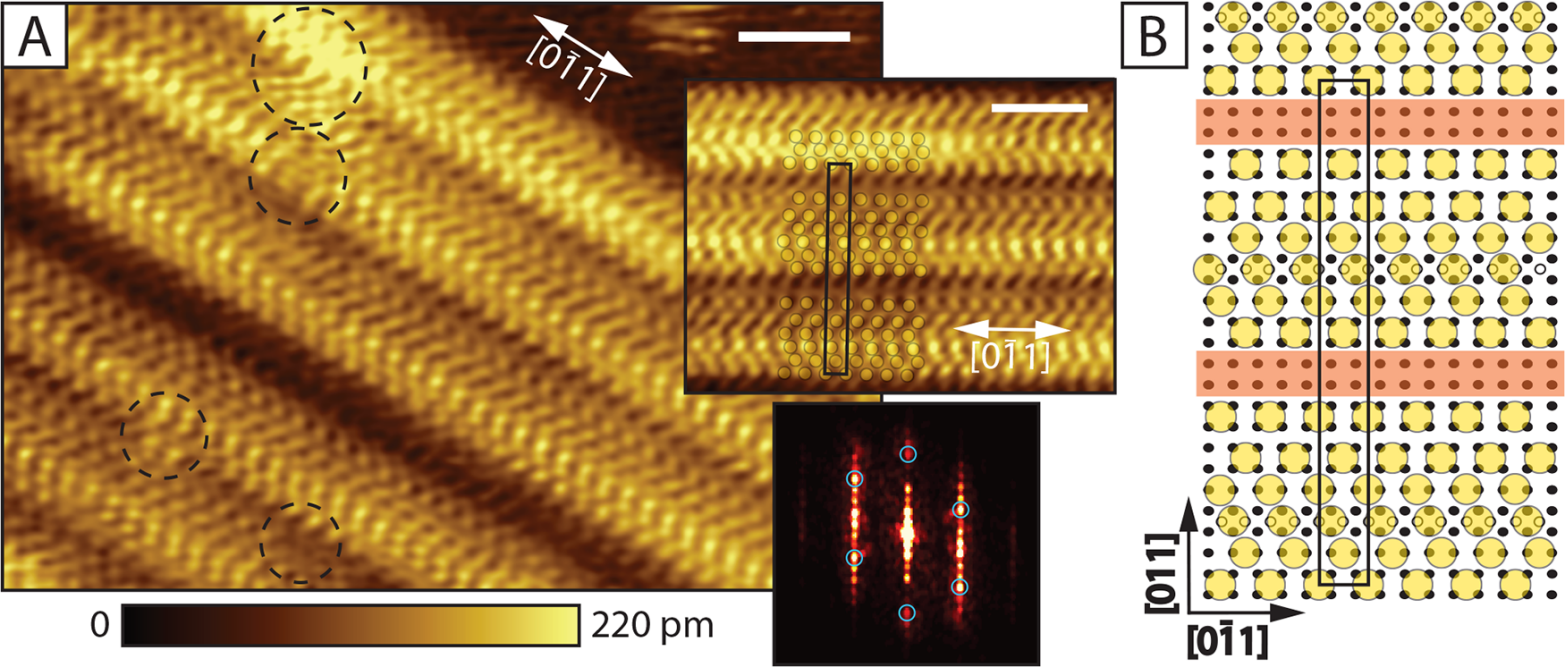
β′ Phase. (A) STM image of the striped β′ phase (set-points: 0.5 V, 80 pA, scale bar: 3 nm). With dashed circles we mark the places where small-scale dynamic effects take place, the ordering is disturbed. On the top inset part of the image is shown, with the model in (B) overlaid on top of the data (scale bar: 3 nm), the unit cell is shown in black. On the lower inset the FFT pattern corresponding to that phase is shown, the hexagonal symmetry is marked with blue circles. Extra spots appear in direction, perpendicular to the stripes, the FFT is aligned with the top inset. (B) Suggested phase model, the *c*(2 × 24) unit cell is marked in black. Large yellow circles mark the position of the S-head, the small black circles indicate the Au atoms in the unreconstructed surface. Small open circles indicate the location of Au adatoms, which arrange in added rows (1 × 12 added row). Orange stripes indicate the locations at which the molecule positions cannot be unambiguously defined.

We begin with the first striped phase variant. As shown in Figure 4(A), the stripe orientation is along the  direction. The β′ phase is a hexagonal arrangement with repeating periodicity perpendicular to the striped direction. This is also clear from the FFT pattern (lower inset), on which extra spots appear perpendicular to the striped rows (upper inset). As for the other phases presented in this paper, there are some dynamic regions, in which the ordering is disturbed. Such places are marked with dashed circles in Figure 4A.

In Figure 4B, we give a possible model of the phase which is also overlaid on top of the STM data inset in (A). The unit cell is of the form *c*(2 × 24), given in Au lattice spacing units. We have applied the same rules in preparing the model as for the planar phases. To account for the height variation, a Au adatom row in placed once in 12 Au rows of the unreconstructed substrate because one molecular row per stripe seems to be elevated with respect to the remaining rows in the stripe. Whether the whole striped structure is formed on top of an elongated Au adatom island is hard to determine as next to the β′ phase, either a disordered phase or a planar phase forms. Therefore, the different electronic contributions from the molecular tails would further modulate the apparent height. In the Supporting Information (Figure S3), we show a height profile for the β′ phase, which suggests that only for the molecular rows modeled on top of Au adatom rows the height is sufficient to account for the 0.2 nm Au(001) monolayer step. The remaining height variation may emerge due to the carbon backbones of the molecules, or the noncomplete lifting of the hex reconstruction underneath the molecules. The latter can also explain the slight “wavy” appearance of some molecular rows in the STM data (see Figure 1A for comparison). The width of the total unit cell of the β′ phase is about 6.2 nm (see the width profile in Figure S3 of the SI), which means that a single stripe of the β′ phase is about 3.1 nm, or very close to twice the width of the hex reconstruction (a profile is also presented in Figure S3 of the SI).

When we look at the overlay of the model on top of the data in Figure 4A, we observe that inevitably deviations occur, either because of the reasons discussed above, or because of slight incommensurability effects or the tolerance in stretching/contracting the Au lattice spacings (see Table 1 in the SI). Nevertheless, our model in 4(B) agrees closely to the data in (A). The large *c*(2 × 24) unit cell that we suggest accounts also for the shift of the striped phase: neighboring stripes are shifted with a single Au lattice position horizontally. The large *c*(2 × 24) unit cell, in combination with some incommensurability effects can account also for the larger trough in Figure 4A, separating neighboring β′ phase regions. Only the regions marked in orange in Figure 4B were left out of the model, as the periodicity within this portion of the stripes cannot be resolved unambiguously. Most likely, this is due to the STM tip radius which is not small enough to resolve the sudden height variation in these molecular rows.

Due to the tolerance in stretching/compressing the Au lattice spacing, the *c*(2 × 24) unit cell may also be a c(2 × 22) unit cell in reality: then 1 Au adatom row would be added each 11 rows of the unreconstructed substrate, for instance. This, in combination with the regions marked in orange in the model, prevents us from giving a precise coverage estimation. However, we can provide an estimation which tends to the lower limit (in this model the lattice parameter is contracted along the  direction, see Table 1 in the SI). The β′ phase coverage derived based on the *c*(2 × 24) unit cell, excluding the orange regions, is θ ∼ 0.27. If we assume an extra molecule located in the orange region of the unit cell, the coverage is then θ = 0.29. Finally, if we assume the a c(2 × 22) instead, the coverage becomes θ ∼ 0.32. This is very close to the coverage of the β phase^55,57,64^ as we pointed out earlier. However, clearly our model deviates from the structure of the archetype, most likely due to the generally lower decanethiol coverage in our experiment.

### The β^″^ Phase

**Figure 5:**
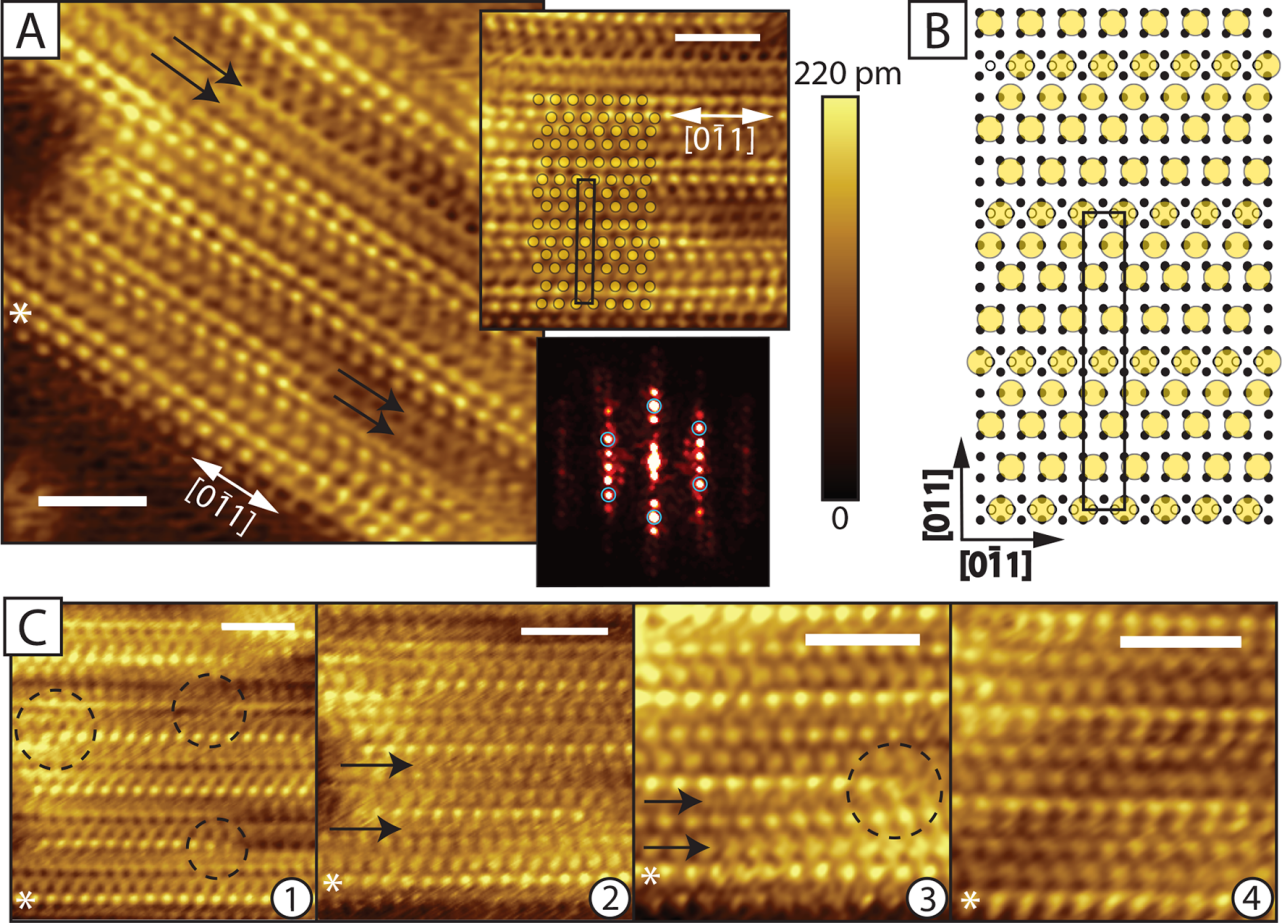
β″ Phase. (A) STM image of the striped β′ phase (set-points: 0.5 V, 80 pA, scale bar: 3 nm). With arrows we mark the places where the molecular rows appear to be of changed periodicity, and thus the ordering departs from the model. On the top inset part of the image is shown, with the model in (B) overlaid on top of the data (scale bar: 3 nm), the unit cell is shown in black. On the lower inset the FFT pattern corresponding to that phase is shown, the hexagonal symmetry is marked with blue circles. Extra spots appear in direction, perpendicular to the stripes. (B) The suggested phase model, the *c*(2 × 14) unit cell is marked in black. Large yellow circles mark the position of the S-head, the small black circles indicate the Au atoms in the unreconstructed surface. Small open circles indicate the location of Au adatoms, which arrange in added rows (1 × 7 added row). (C) Short time sequence of images taken at approximately the same area as in (A) (all scale bars: 3 nm). A white star indicates the same molecular row on each panel, as well as in (A). With arrows we mark the rows where the molecule positions seem to be less well-defined, it is possible that molecules switch between available adsorption sites. Dashed circles indicate regions with “wavy” appearance, which remind us of the hex reconstruction. Panels 1–4 were taken, respectively, about 1 h, 40, 30, and 20 min before the image in (A).

Another striped variant that was measured is presented in Figure 5A, with a simplified model presented in Figure 5B. Compared to the β′ phase, we observe more frequently regions that deviate from the main ordering (such molecular rows are marked with arrows). Moreover, observing this phase throughout time lead to structural changes which also suggests a dynamic phase.

Again, we deal with a hexagonally distorted pattern with stripes running along the  direction. Analogously to Figure 4, we provide a phase model in (B), which is overlaid on top of the STM data in the upper inset in (A). The FFT pattern (lower inset) in (A) is similar to the β′ striped phase: extra spots appear in a direction perpendicular to the molecular rows. However, the periodicity perpendicular to the stripes of the β″ phase is clearly different, which leads to a separate model for this phase.

Width and height profiles of the β″ phase are given in the SI (see Figure S3). Only one molecular row per stripe is elevated enough to account for the 0.2 nm Au(001) monolayer step. Therefore, we again model these molecular rows in the bridge positions on top of Au adatom rows. The remaining height variations we can again assign to the contribution of the molecular tails, as well as the nonfull lifting of the hex reconstruction underneath. Similar shift of neighboring stripes doubles the unit cell periodicity in the ⟨011⟩ direction: instead of *c*(2 × 7), the unit cell is of the form *c*(2 × 14). The large lattice parameter of the suggested *c*(2 × 14) unit cell is 4.28 nm. Therefore, the width of a single stripe of the β″ phase is about 2.14 nm, which is larger than the hex reconstruction width and smaller than twice the hex reconstruction width. Therefore, we do not deal with a pattern that would fit nicely on top of the partially lifted hex rows. That can explain the lack of large scale ordering in the phase: frequently rows of altered periodicity appear, marked with arrows in Figure 5A. Of course, it is also possible that in these rows the molecular coverage is different, or partially the Au lattice underneath gives contribution. In such rows, the molecular position is harder to determine, the molecules may also switch between available adsorption sites. Based on the *c*(2 × 14) model, the estimated density of the β″ phase is θ = 0.29, again somewhat lower than the archetype β phase.

We note that the β″ phase has experienced some dynamic rearrangements in time. The panels in Figure 5C show a few images taken prior to the image in (A). At first, the height distribution is a lot steeper: only one molecular row is of larger apparent height (panel 1), while later the height distribution from molecular row to molecular row becomes more gradual (panel 3, panel 4). In the time sequence, we have also marked regions with “wavy” appearance (dashed circles) to demonstrate the resemblance to the hex reconstruction features. And finally, we again mark molecular rows of higher periodicity, in which the molecular position cannot be determined unambiguously. That may be a sign of fast switching effects: the molecules change rapidly their location between adsorption sites which are close energetically. After the structure in (A) was established, the phase did not go through drastic further changes. It is, of course, possible that the STM tip itself has experienced changes, leading to altered tunneling conditions, and thus the change in the apparent height of the phase. Moreover, the tip itself may be responsible for altering the phase in situ. Nevertheless, such changes were not observed for the β′ phase, which makes us reluctant to accept the dynamics scenario.

In summary, we presented two striped decanethiolate phases: the β′ and the β″ phase. While they are similar in arrangement to the previously reported β phase, they do not have the same structure. In fact, the originally reported β phase with *c*(2 × 8) structure grows on top of elongated Au adatom islands with 1 row in 4 Au adatom rows missing.^57^ Grumelli et al. have reported a similar phase,^54^ but even that phase is clearly of different structure compared to the measurements of Poirier. While the phases reported by Grumelli et al. and Poirier share the similarity that they form on top of elongated Au adatom islands, if we look close to the data of Schweizer et al.,^52^ the β phase reported is growing within the same Au layer as neighboring domains of the square α phase. Therefore, clearly there are discrepancies which need to be still resolved. We suggest that the striped phases that we observed belong to the same family of β-like phases, however they cannot be modeled with the same unit cell and therefore we try to distinguish this by using the alternative labeling. Because the apparent height measured in our experiments is too low to assume that the striped phases are growing on top of elongated Au islands, we assume that they are structurally more similar to the results of Schweizer et al.^52^ We note that we have also observed striped arrangements which seem to be elevated enough to account for the Au(001) monolayer step, for instance in Figure 1B. However, these were more rare and still shared the hexagonal arrangement reported earlier, and therefore, were not the focus of our analysis. The coverage estimations for the β′ and the β″ phases is somewhat lower (∼0.29) compared to the coverage reported earlier for the thiol striped phases. That is most likely also the reason for the different unit cells and the dynamic effects observed for the β″ phase. Of course, we must also keep in mind that we are comparing results from different studies that use different chain length thiols, which further complicates the task of coming up with a universal classification of the alkyl thiol phases on Au(001).

## Computational Results

**Figure 6:**
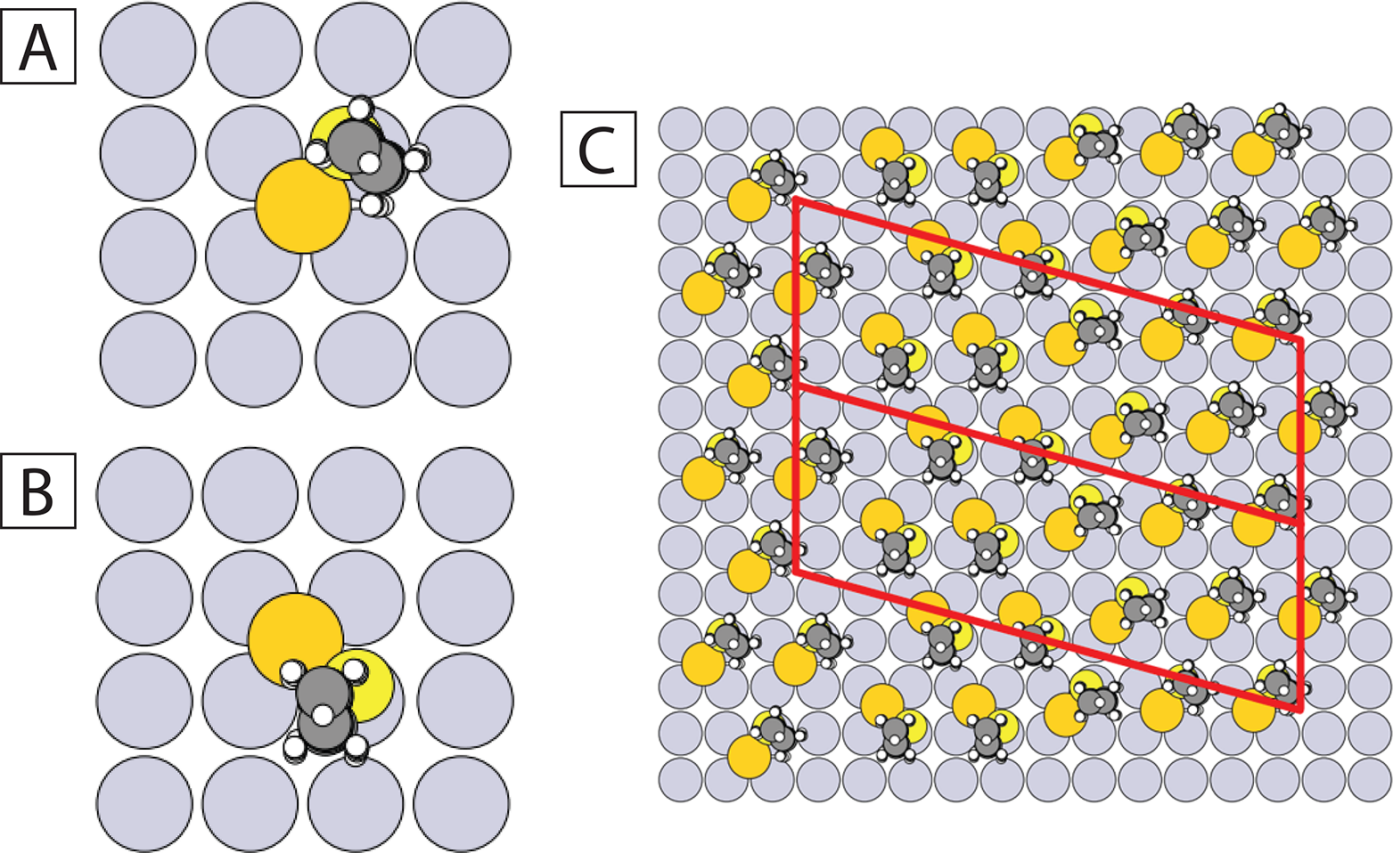
(A,B) Two possible atomic models of configurations of adsorption on Au(001) for Au–SR complexes. Color code: Au (bottom) = gray, Au (top) = dark yellow, S = light yellow, C = dark gray, H = white. (C) Proposed model for the φ phase. Two consecutive unit cells are marked in red.

In our previous study, it was shown that the most likely diffusing moieties on Au(001) are Au–SR complexes in which a single decanethiol molecule binds to a Au adatom and Au–SR complexes continuously diffuse on top of the surface at low coverage.^81^ Thus, we considered only Au–SR complexes on the surface in the following model. First, we found possible adsorption sites for the Au–SR complex. For this, all possible adsorption sites on the surface were tested. Figure 6 shows the two most stable configurations for the Au–SR complex. The Au adatom is located in a hollow site and the S atom is located next to one of the four gold surface atoms. The S atom binds to these two neighboring gold atoms in both configurations. The difference between the two configurations is that in the first case, the tail of decanethiol molecule is above the surface atom (see Figure 6A), while in the second case, the tail of decanethiol molecule is between the two surface atoms (see Figure 6B). The total energy difference between these two configurations is only a few meV. The activation barrier for diffusion of Au–SR complex is 285 meV.^81^

Next, we calculated the interaction energy of the two Au–SR complexes as a function of the distance between them (see Table 1). An analysis of values of the interaction energy presented in Table 1 shows that the configuration will have the lowest energy when each molecule has two-third neighbors and four fourth neighbors. Thus, each molecule should have six neighbors, which agrees with the hexagonal symmetry observed for the φ phase (see Figure S4A in the SI). The energy of the resulting configuration will be the lowest with the largest number of molecules on the surface. However, it is difficult to obtain such a configuration (see Figure S4B from the SI) in an experiment, because in this case, there should be only one growth center. In reality, many growth centers are formed in the process of self-organization around which the growth of structures occurs. Then these structures can coalesce. In this case, defects in the form of empty zones can form at the structure boundary. Taking this fact into account, we performed simulations of the evolution of different initial configurations which had different empty zones. These are all shown in Figure S4 in the SI.

When there are empty islands in a monolayer of Au–SR complexes, then the Au–SR complexes form a disordered structure after a short period of time due to diffusion at room temperature. In this case, the total energy slightly increases and fluctuates around the average value of −334 meV/mol. It was surprising to find a completely opposite behavior of the system for the case, when the empty zone looks like a narrow line (see Figure S4D). Only rotations of Au–SR complexes near an empty narrow zone are possible, and there is no diffusion. The total energy does not change. Therefore, a narrow empty line will stabilize the structures that are formed by the Au–SR complexes.

In summary, based on DFT calculations and kMC simulations, on the one hand, the system must be compact and have the maximum number of Au–SR complexes in order to have the lowest energy. On the other hand, for its stability, the system must have a narrow empty line zone. Moreover, there can be two types of Au–SR complexes in the system. Considering these results and analyzing the experimental STM images, we have built a model of the φ phase with Au–SR complexes on Au(001) (see Figure 6C). The difference between the suggested models for the φ phase are only marginal, showing the relevance of continuing to address the Au–SR complex also on Au(001).

## Disordered Phase Regions

Finally, we wish to pay attention to the last common part of the decanethiol SAM that we have observed: the disordered phase regions. In such parts of the SAM no defined phase forms, the molecules are also mobile. Therefore, considering the relatively low coverage of the phases we looked at, we assume that the coverage in the disordered regions is quite low, but still higher than the disordered decanethiolate monolayers we measured in our previous work,^81^ otherwise it would be possible to measure the exposed Au lattice.

**Figure 7:**
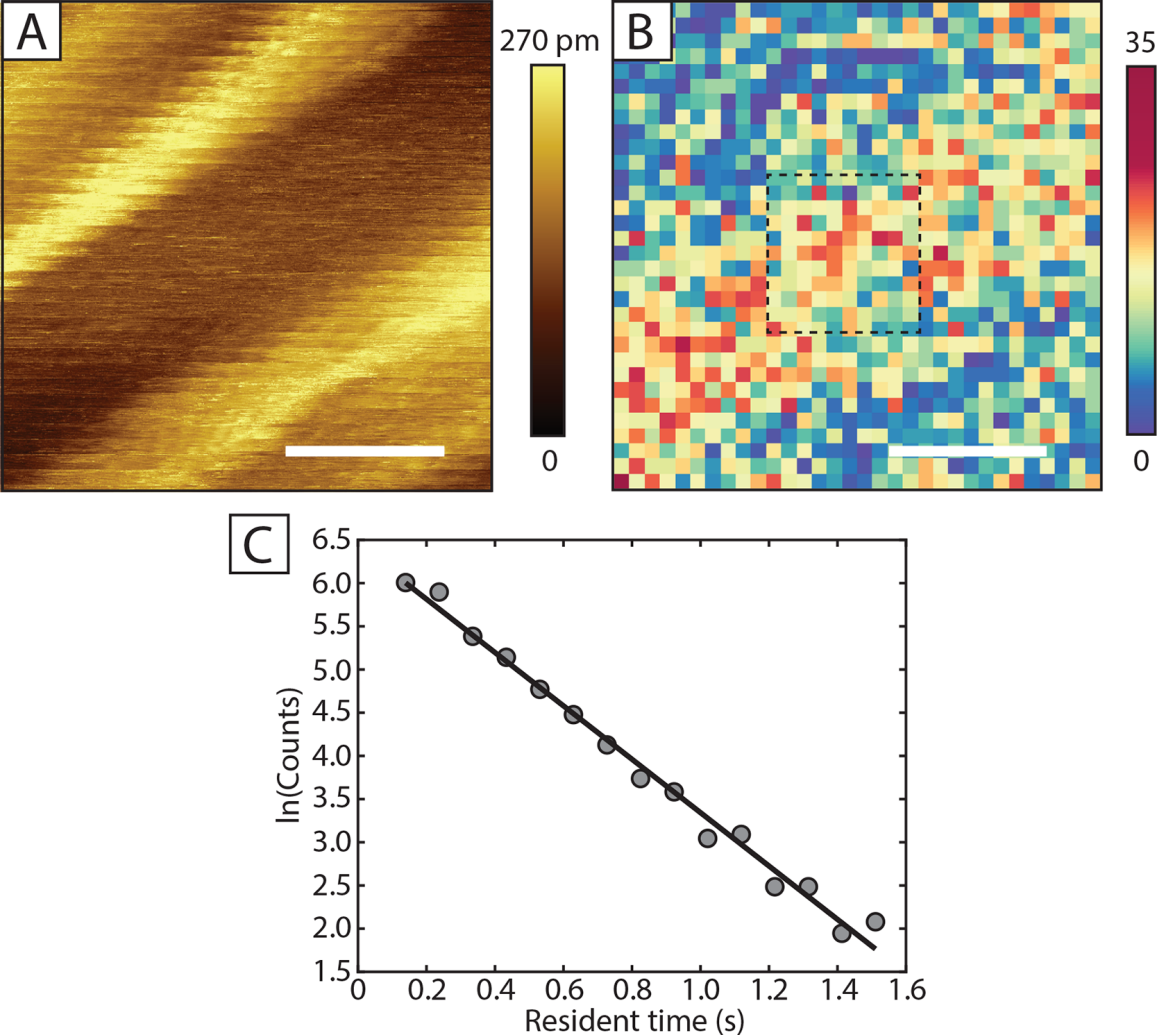
(A) *I*(*t*) grid (15 × 15 nm^2^, topography), set-points: 500 mV, 80 pA, scale bars in (A,B) correspond to 5 nm. Spectroscopy locations are set at 0.5 nm apart. (B) *I*(*t*) heatmap of the detected switches. The dashed square indicates the area from which the distribution of residence time in (C) is taken. (C) Distribution of residence times between the switches. The linear fit corresponds to the formula *y* = −3.0892*x* + 6.4324.

To investigate further the disordered phases, we have performed *I*(*t*) spectroscopy: at each spectroscopic location we disable the feedback loop and record an *I*(*t*) trace for about 8 s. That way, we can compare the dynamic signatures of different locations on the sample. A measurement is presented in Figure 7. In (A), a wide disordered region can be found between two striped regions. Because of drift, the topography of the *I*(*t*) grid looks a bit distorted. However, if looking close, the ordering in the stripes can be seen. To compare the stripes versus the disordered region, we show a so-called heatmap of the switching phenomena detected in Figure 7B. A switching event was detected by looking at the local mean of the traces. If a threshold is passed as the local mean changes and if the segment of the given local mean is long enough, a switch was detected. All traces were analyzed with the same mean change threshold (5 pA) and length criterion (100 ms), which lead to the switch count in the heatmap. Clearly, more switches are detected at the disordered region if compared to the ordered striped regions at the sides. This suggests that the disordered decanethiolate regions are more dynamic. The molecules are still diffusing on the surface, but the coverage is most likely too low, while at the stripes, the molecules are ordered, and the tip would measure a more stable current over time. Of course, switching due to the wagging of the molecule tails, or the movement of adsorbates is possible. As a step further, we can say a bit more on the nature of the switching phenomena in the disordered region. In Figure 7C, a histogram plot of the resident times between switches in the center of the heatmap is presented. Clearly, there is a linear dependence which suggests a stochastic switching process,^22,83−85^ or the switching is random. From the value of the slope of the linear fit (3.089 Hz), we learn that about 3 switching phenomena per second take place.

## Conclusion

We studied decanethiolate phases on Au(001), deposited via the solution method. Based on our findings, the SAM coverage is lower compared to other reports. That may explain the appearance of phases which were not previously reported: the planar φ and φ′ phases, as well as the structurally similar to the previously reported thiol β phase striped β′ and β″ phases. Models for these phases were suggested. The φ phase is a hexagonal phase with a  overlay structure, rotated at about 5° with regard to the  direction. The φ′ phase is also hexagonal and has a *c*(2 × 3) unit cell with respect to the unreconstructed Au(001) surface. For the φ phase we also suggested an alternative model, in which the bulding blocks are not bare thiol molecules, but Au–SR complexes, demonstrating the relevance in continuing to address these moieties also on Au(001). The β′ and β″ phases have large unit cells w.r.t. the unreconstructed Au(001) surface: *c*(2 × 24) and *c*(2 × 14), respectively. The doubling of the corresponding *c*(2 × 12) and *c*(2 × 7) unit cells accounts for the shift of neighboring stripes with a Au lattice position. In the modeling of these phases we have assumed the presence of Au adatom rows because the apparent height of the stripes is not sufficient to assume that they grow on top of elongated Au adatom rows. We also looked at disordered SAM regions and performed *I*(*t*) spectroscopy. Most likely, in these regions the SAM coverage is too low in order to form a denser phase. These disordered phases are also more dynamic compared to the ordered striped phases nearby. The current switching recorded at these regions is stochastic: most likely the molecules still diffuse randomly on the surface.

All of the phases that we measured are of somewhat lower coverage compared to previous reports. To continue expanding the family of known thiol phases on Au(001), it is important to perform studies in which the thiol concentration in the solution is varied, as well as the exposure time, and the chain length of thiols. Studies which are fully performed in UHV conditions would be even more valuable, as then the formation mechanisms can be studied in greater detail, and the coverage can be monitored *in situ*. That would be especially important for understanding the interaction of growing molecular phases with the Au adatoms expelled during the lifting of the hex reconstruction.

## Supporting Information Available

Additional measurements and analysis; model details; scan profiles; simulations; XPS (PDF)
