# Supplementary material for: Self-Assembled Decanethiolate Monolayers on Au(001): Expanding the Family of Known Phases
Source: Langmuir. 2022 Aug 11;38(33):10202–15. doi: 10.1021/acs.langmuir.2c01356 (PMC9404544; doi:10.1021/acs.langmuir.2c01356)
Supplement: Supplementary file 1 — la2c01356_si_001.pdf [file la2c01356_si_001.pdf]

# Self-assembled decanethiolate monolayers on Au(001): expanding the family of known phases

**(11 pages, 1 table, 5 figures)**

Martina Tsvetanova,*,† Alexey G. Syromyatnikov,‡ Thomas van der Meer,† Arie van Houselt,† Harold J. W. Zandvliet,† Andrey L. Klavsyuk,‡ and Kai Sotthewes*,†

†*Physics of Interfaces and Nanomaterials, MESA+ Institute for Nanotechnology, University of Twente, Post Office Box 217, 7500AE Enschede, the Netherlands*

‡ *Faculty of Physics, Lomonosov Moscow State University, Moscow 119991, Russian Federation*

E-mail: m.tsvetanova@utwente.nl; k.sotthewes@utwente.nl

# Phase models details and limitations

In this research an RHK scanning probe microscope was deployed. The system uses a beetle-type scanner which explains the different orientation of the crystallographic directions on the various STM images presented. Another limitation due to the scanner is the potentially position-dependent stretching/contracting of the measured in-plane lattice parameters. We have performed calibration experiments with a HOPG sample and have found out that with time at a given location, the measured lattice parameters become closer and tend to the expected values. While at the beginning an error up to 20% may be expected, later on the error becomes smaller. Because of the slow relaxation in time, it was not possible to perform full position-dependent calibration. Nevertheless, because all of the images presented were taken after the initial large drift effects were gone, we expect errors less than 20%. This is confirmed by the stretching measured for the width of the hex reconstruction (see the profiles given at the end of the SI), which is at most stretched with 14%, as deduced from the high resolution images which were obtained. In the out-of-plane direction, the apparent height measured showed to be less than expected, a calibration coefficient of about 1.3 was found. This coefficient was already taken into account when plotting the profiles in the last section of this SI.

Because there was no clean Au(001) surface exposed in the close vicinity of the molecular phases, the phases were modeled on top of a square grid which is to represent the unreconstructed Au(001) surface. We took the decision to use tolerances in the $x$ and $y$ directions of the grid up to 10%. Table S1 shows the lattice parameter values deployed for the models in this paper. The values are given as the percentage of the expected in-plane Au(001) lattice parameter of 0.288 nm. These are the values for which we obtained a reasonable agreement between data and model overlay. Of course, especially to account for the complex height variation in the striped phases, more sophisticated models would be required, which take into account the orientation and contribution of the molecular tails. Also, slight incommen-

surabilities cannot be easily modeled with this approach. Note, for instance, that changing the tolerances in the table may lead to some deviations in the unit cell labels. This is more relevant for the striped phases as they have a quite large unit cell. For instance, the $\beta'$ phase can be modeled in a similar way as shown in Figure 4(B) in the main text. If we use a lattice parameter in the $y$-direction of 100% (instead of 90%), for example, the unit cell will become a $c(2{\times}22)/(1{\times}11$ added row), instead of $c(2{\times}24)/(1{\times}12$ added row), and the overlap with the experimental data would be still reasonable (some molecule rows only shift slightly from hollow to a bridge site). Therefore, the models we present must be seen as approximations which can only be confirmed with an extensive DFT study, given the unit cells always contain quite many Au atoms.

**Table S1:** Table with the Au(001) in-plane lattice parameter values used in the Au grids of the phase models. The values are expressed as a percentage of the expected unreconstructed-Au(001) in-plane lattice parameter of 0.288 nm.

| **Phase** | **Model** | **x[%]** | **y[%]** |
|---|---|---|---|
| $\varphi$ | Figure 2(D) | 100 | 100 |
| $\varphi'$ | Figure 3(B) | 105 | 105 |
| $\varphi''$ | Figure S.2(B) | 105 | 105 |
| $\beta'$ | Figure 4(B) | 105 | 90 |
| $\beta''$ | Figure 5(B) | 105 | 108 |

# Additional STM data

## Phase domains

At first we present two domains of the $\varphi$ phase, measured close to each other, shown in Figure S1(A). Clearly a rotation of both $\pm5^\circ$ with respect to the $[0\bar{1}1]$ direction is observed. Due to the square symmetry of the unreconstructed Au(001) substrate, most likely two more domains of this phase exist.

In Figure S1(B) we show a small portion of the $\varphi'$ phase (encircled with a dashed shape) close to another planar phase. Below we present a model of this phase too. Due to its

similarity to the $\varphi'$ phase, we label it as the $\varphi''$ phase.

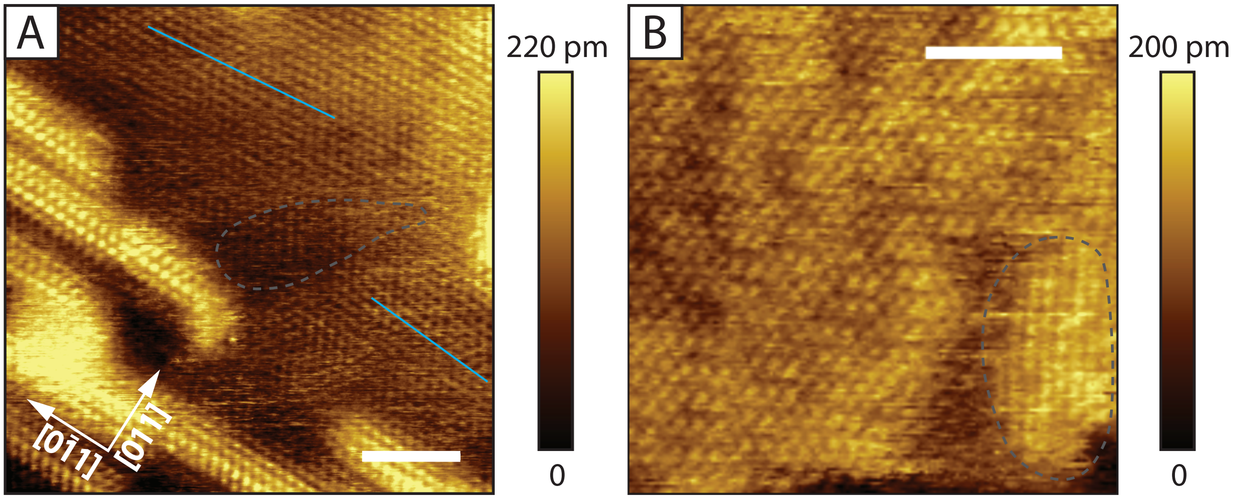


**Figure S1:** Phase domains. (A) Two rotational domains of the $\varphi$ phase (scale bar: 5 nm). The orientation of the phase is marked with blue lines. The transition region between the two domains is marked with a dashed shape. (B) A small region of the $\varphi'$ phase (encircled with a dashed shape) next to another planar phase, modeled below in the SI (scale bar: 5 nm).

STM data for the $\varphi''$ phase is shown in Figure S2(A). The FFT pattern of a defect-free part of the $\varphi''$ phase is given as an inset. Again, a hexagonal structure is observed. The FFT looks similar to the one in Figure 3(A) from the main text, again a centered cell can be selected. For the model in Figure S2(B), again mostly bridge sites were assumed, and less 4-fold hollow sites. This choice was made in order to account for the lack of striking height variations in the STM data. Next, a commonly observed feature in Figure S2(A) is that the molecular rows deviate from a straight line and sometimes are closer to each other, as shown with arrows. That was taken into account in our model by shifting some of the molecules with half of the Au lattice parameter in every second row starting from top to bottom. Just as the previously discussed phases, there is no large scale homogeneous repetition of the modeled pattern. This may be due to phase incommensurability effects.

The resultant phase structure for the $\varphi''$ phase looks strikingly similar to the model in Figure 3(B) from the main text (when rotated over 90°, check the analogously selected centered cell in Figure S2(B)): apart from the shifted molecules shown in darker color, the

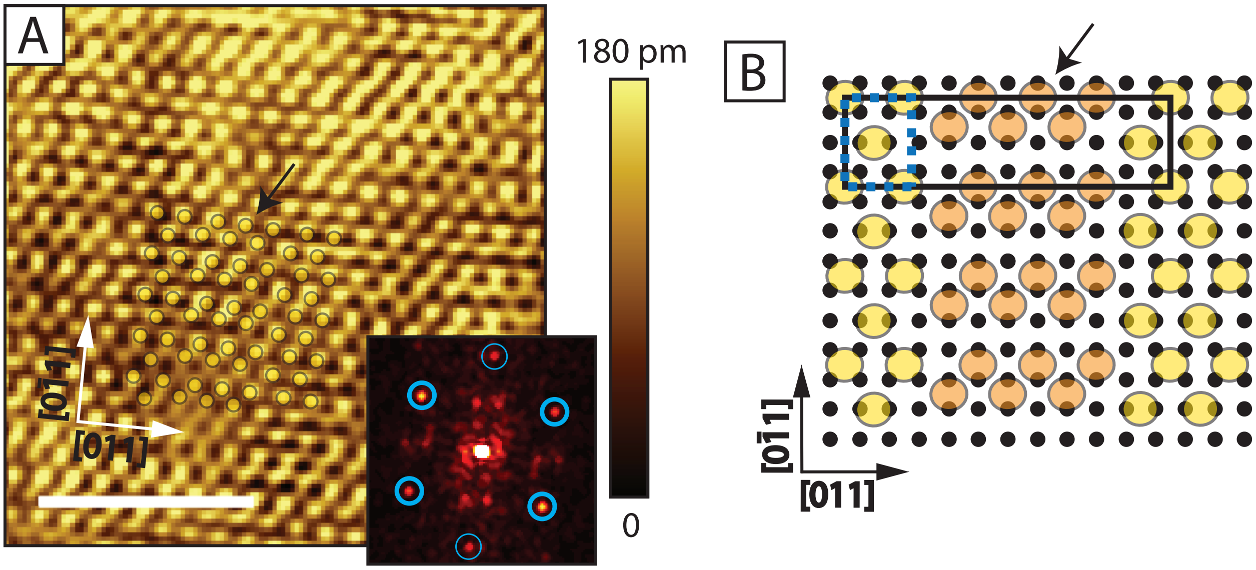


**Figure S2:** (A) STM image of the $\varphi''$ phase. An FFT is shown as an inset. The circles indicate the hexagonal pattern, while the circles with thicker stroke indicate the centered cell selected. The model in (B) is overlaid on top of part of the data. (B) The suggested model for the $\varphi''$ phase. Large circles indicate the location of the molecular S-heads, small black circles indicate the Au(001)-(1×1) topmost layer. The centered rectangular unit cell is marked with a blue dashed rectangle. The large unit cell featuring also the shifted molecules, colored in orange, is shown with a black rectangle. Arrows in (A) and (B) indicate locations where molecular rows are shifting close together.

structure is the same. This, in combination with the similar FFT patterns, suggests that the $\varphi''$ phase is structurally and energetically very close to the $\varphi'$ phase. Furthermore, it is possible that the difference in the molecular tails-tip interactions due to the orientation of these phases contributes to their different appearance, while they are quite analogous to each other and simply rotational domains of the same phase.

## Phase profiles

Profiles of the striped $\beta'$ and $\beta''$ phases are shown in Figure S3(A-D). From the height profiles we learn that only a single molecular row per stripe is of high enough apparent height to account for the monolayer step expected (0.2 nm). That is why, these rows were modeled on top of Au-adatom rows, as discussed in the main text. The width of the phases is also deduced from the width profiles. The $\beta'$ phase is 6.2 nm wide, a single stripe of this phase is 3.1 nm

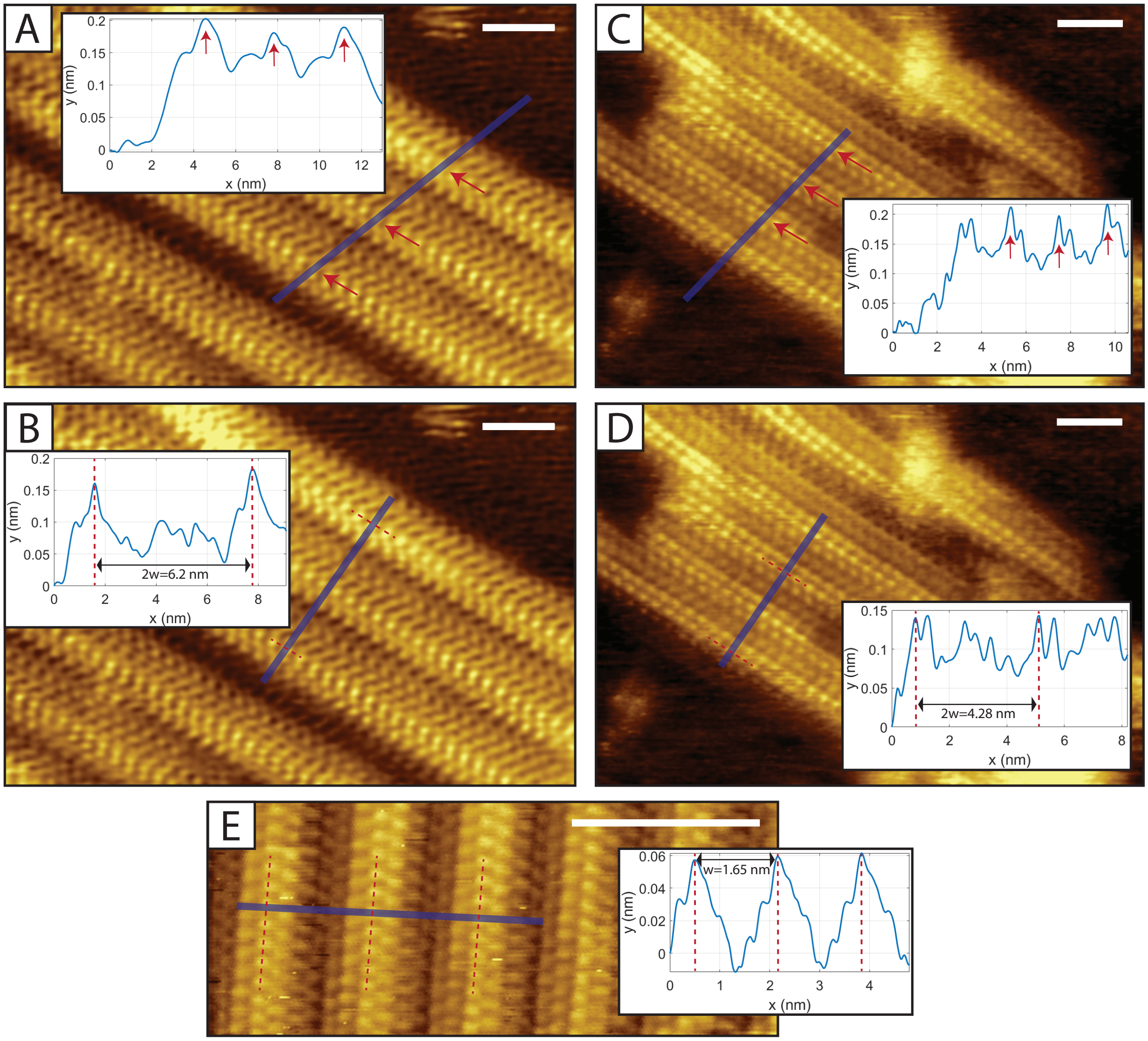


**Figure S3:** Phase profiles. All scale bars correspond to 3 nm. (A) Height profile of the $\beta'$ phase. The profile graph is shown in the inset. With red arrows we mark the highest molecular rows, at which the height would be sufficient to account for the Au(001) monolayer step of 0.2 nm. (B) Width profile of the $\beta'$ phase. The profile graph is shown in the inset. The width of the phase (the whole unit cell) is 2w=6.2 nm. A width of a single stripe is w=3.1 nm. (C) Height profile of the $\beta''$ phase. The profile graph is shown in the inset. With red arrows we mark the highest molecular rows, at which the height would be sufficient to account for the Au(001) monolayer step of 0.2 nm. (D) Width profile of the $\beta''$ phase. The profile graph is shown in the inset. The width of the phase (the whole unit cell) is 2w=4.28 nm. A width of a single stripe is w=2.14 nm. (E) Profile across the hex reconstruction. The profile graph is shown as an inset. The width of a hex stripe is w=1.65 nm.

wide. The $\beta''$ phase is 4.28 nm wide, a single stripe of this phase is 2.14 nm wide. A profile across the hex reconstruction is shown as well in (E). Having in mind the width of the hex reconstruction (1.65 mm), we learn that a single stripe of the $\beta'$ phase contains approximately twice the width of the hex reconstruction. A single stripe of the $\beta''$ phase is wider than the hex reconstruction, and less wide compared to two hex reconstruction stripes. The expected width of the hex reconstruction is 1.44 nm (perpendicular to the stripe direction, 6 atoms of the hex overlayer must fit on top of 5 atoms of the underlying unreconstructed substrate). The fact that we measure a slightly wider hex stripe demonstrates the limitations of our beetle scanner. That is why, we made the decision to use the tolerances as given in Table S1 when modeling the phases.

## Computational results

In this section we present the results from the simulations addressed in the computational section from the main text.

The initial molecular configurations that we considered are shown in Figure S4, we have used only one of the equivalent Au–SR configurations on top of Au(001). The configuration in (A), (C), and (D) have the lowest total energy. The total energy of these configurations is -415 meV/mol, -415 meV/mol, and -416 meV/mol, respectively. The total energy of the configuration shown in (B) is higher and equal to -386 meV/mol. Note that in the case of a random arrangement of Au-SR complexes on the surface, the total energy varies from -285 meV/mol to -214 meV/mol.

## XPS results

The chemical nature of the decanethiol molecules on Au(001) was assessed by X-ray photoelectron spectroscopy, XPS (see Fig. S5). The S2p region of the XPS spectrum is shown. Usually, the S2p3/2 core level peak for SAMs of thiols on Au can be decomposed into three components; (i) S1 at 161 eV binding energy (BE), associated with atomically adsorbed

sulfur, (ii) S2 at 162 eV BE related to S atoms chemisorbed on the metal surface through a thiolate bond in the thiol-Au interface and (iii) S3 at 163.5 eV BE, indicating physisorbed species.[1–3] In Fig. S5, two of these components are visible: S2 and S3; The S2p3/2 peak is located at 161.7 eV BE and the S2p1/2 peak at 162.9 eV BE. This is in good agreement with previous measurements of hexanethiol adsorption on Au(100).[1] The separation between the S2p3/2 and S2p1/2 peak is fixed to 1.2 eV which is the spin-orbit doublet separation. The S3 component is located near 163.5 eV BE. This component is very broad and low in intensity, demonstrating the dominance of chemisorbed species over the physisorbed ones.

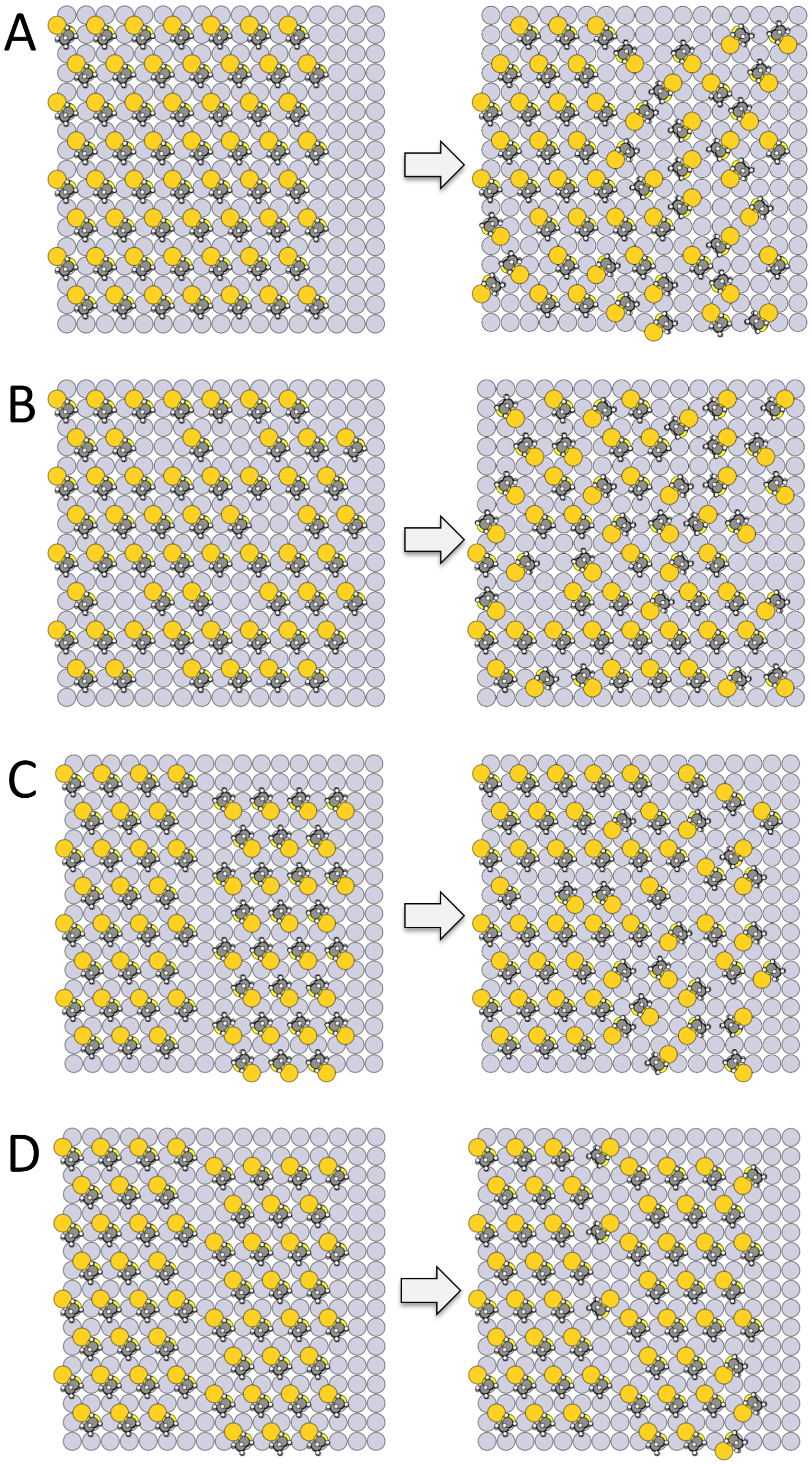


**Figure S4:** Different initial (left) and corresponding final (right) configurations of Au-SR complexes at high coverage on Au(001).

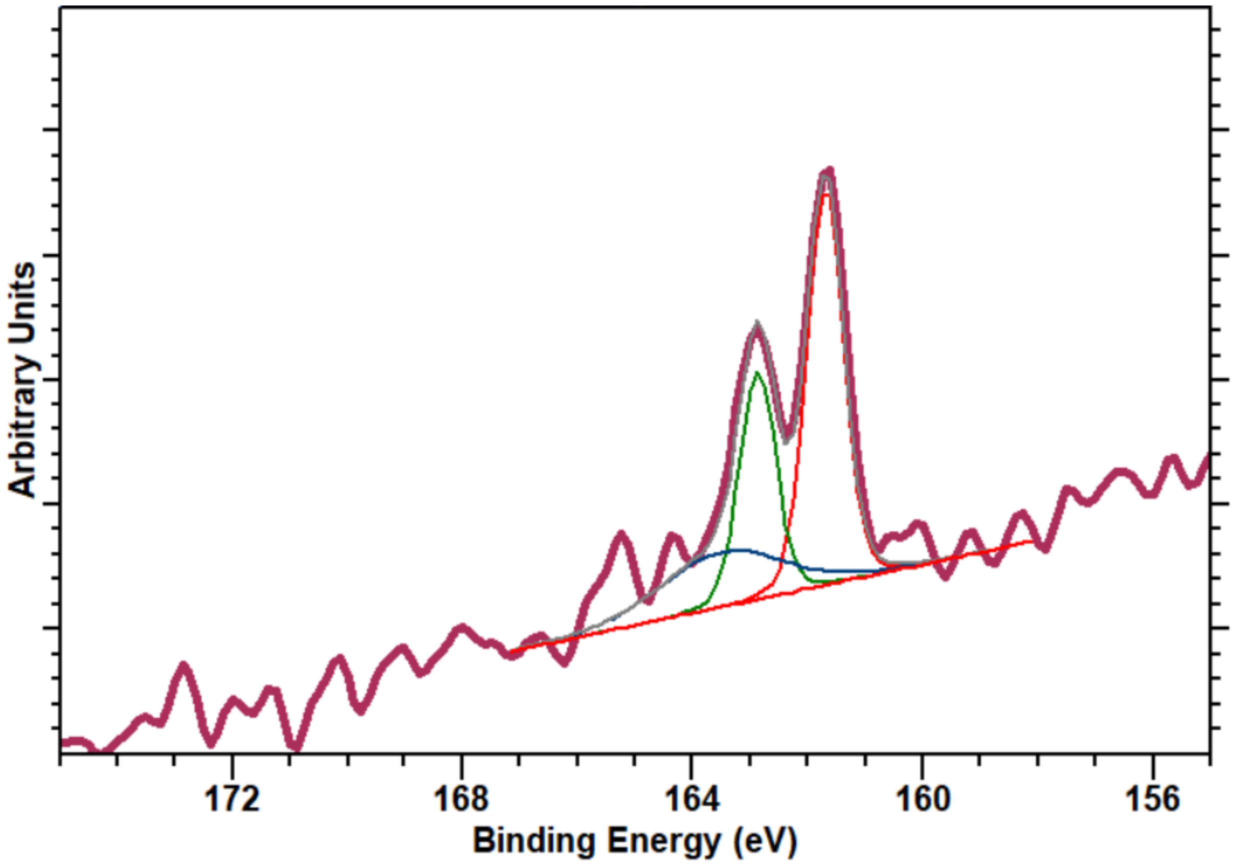


**Figure S5:** S2p spectrum (thick, magenta) of a freshly prepared decanethiol SAM on Au(001). The best fit (gray line) and the elemental components are shown (S2 doublet, green and red, and S3, blue).

## References


(1) Grumelli, D.; Cristina, L. J.; Maza, F. L.; Carro, P.; Ferrón, J.; Kern, K.; Salvarezza, R. C. Thiol adsorption on the Au(100)-hex and Au(100)-(1×1) surfaces. *The Journal of Physical Chemistry C* **2015**, *119*, 14248–14254.

(2) Vericat, C.; Vela, M. E.; Corthey, G.; Pensa, E.; Cortés, E.; Fonticelli, M. H.; Ibañez, F.; Benitez, G. E.; Carro, P.; Salvarezza, R. C. Self-assembled monolayers of thiolates on metals: A review article on sulfur-metal chemistry and surface structures. *RSC Advances* **2014**, *4*, 27730–27754.

(3) Waske, P. A.; Meyerbröker, N.; Eck, W.; Zharnikov, M. Self-assembled monolayers of cyclic aliphatic thiols and their reaction toward electron irradiation. *Journal of Physical Chemistry C* **2012**, *116*, 13559–13568.
